# Skeletal Malocclusion: A Developmental Disorder With a Life-Long Morbidity

**DOI:** 10.14740/jocmr1905w

**Published:** 2014-09-09

**Authors:** Nishitha Joshi, Ahmad M. Hamdan, Walid D. Fakhouri

**Affiliations:** a School of Public Health, University of Texas Health Science Center, Houston, TX 77054, USA; b Department of Orthodontics, University of Jordan, Amman, Jordan; c Department of Diagnostic and Biomedical Sciences, Center for Craniofacial Research, School of Dentistry, University of Texas Health Science Center, Houston, TX 77054, USA

**Keywords:** Skeletal malocclusion, Micrognathia, Retrognathia, Prognathia, Late-onset diseases

## Abstract

The likelihood of birth defects in orofacial tissues is high due to the structural and developmental complexity of the face and the susceptibility to intrinsic and extrinsic perturbations. Skeletal malocclusion is caused by the distortion of the proper mandibular and/or maxillary growth during fetal development. Patients with skeletal malocclusion may suffer from dental deformities, bruxism, teeth crowding, trismus, mastication difficulties, breathing obstruction and digestion disturbance if the problem is left untreated. In this review, we focused on skeletal malocclusion that affects 27.9% of the US population with different severity levels. We summarized the prevalence of class I, II and III of malocclusion in different ethnic groups and discussed the most frequent medical disorders associated with skeletal malocclusion. Dental anomalies that lead to malocclusion such as tooth agenesis, crowding, missing teeth and abnormal tooth size are not addressed in this review. We propose a modified version of malocclusion classification for research purposes to exhibit a clear distinction between skeletal vs. dental malocclusion in comparison to Angle’s classification. In addition, we performed a cross-sectional analysis on orthodontic (malocclusion) data through the BigMouth Dental Data Repository to calculate potential association between malocclusion with other medical conditions. In conclusion, this review emphasizes the need to identify genetic and environmental factors that cause or contribute risk to skeletal malocclusion and the possible association with other medical conditions to improve assessment, prognosis and therapeutic approaches.

## Introduction

Disorders of the head and face are very common birth defects in all racial populations, and can appear as isolated phenotype or as part of a syndrome. The prevalence of craniofacial anomalies varies among different ethnicities based on genetic background, geography, socio-economical status and environmental factors. Because of the structural complexity of the craniofacial region, variations in genetic and environmental factors may have a profound effect on development, and could lead to congenital birth defects. Cleft lip and palate is one of the most common birth defects with the highest prevalence of 1 in 500 live births in Asian population [1]. Skeletal malocclusion is another common birth defect that occurs due to the distortion of the maxillary and/or mandibular development that will have a huge impact on the positioning, alignment and health of the primary and permanent teeth. Micrognathia, a small mandible or maxilla, is the most common cause of skeletal malocclusion with a prevalence of 1/1,500 live births [2], and is frequently associated with other skeletal abnormalities, cleft palate and tongue deformaties (glossoptosis). Micrognathia occurs as an isolated form or as part of 468 syndromic disorders according to Online Mendelian Inheritance in Man (OMIM) database. It has been reported that all patients with micrognathia are also affected with retrognathia (abnormal posterior positioning of the mandible or maxilla relative to the facial structure) due to the small size and growth pattern [3]. On the other hand, macrognathia is characterized by the overgrowth of the mandible or maxilla above the normal values where the manifestation becomes more prominent at the peak of jaw growth around the age of 12.2 years in females and 14 years in males [4].

Sonographic detection used for prenatal diagnosis of isolated micrognathia (manifestation of class II malocclusion) is normally disparate from the actual natal outcome in the large majority of cases. More than 90% of fetuses diagnosed with isolated micrognathia by 3D ultrasound represented additional deformities with clefting of soft palate being the most common anomaly (73% of micrognathic cases) [2]. This is due to the small size of the mandible that causes the tongue to stick to the roof of the mouth and prevent the appropriate downward vertical growth, elevation and fusion of the secondary palatal shelves. The clefts of the soft and hard palate in these cases have the characteristic feature of the U or V shape clefting indicating a complete obstruction of secondary palatal development. Other studies have argued that isolated micrognathia is a harbinger of severe diseases in humans [5]. About one-third of children diagnosed with micrognathia also have mild to severe developmental delay [2]. However, a genetic test for many hereditary diseases with micrognathia such as Pierre Robin sequence, isolated micrognathia, agnathia-otocephaly complex, Catel-Manzke syndrome and cerebrocostomandibular syndrome cannot be ordered because the etiological factors have not been demarcated. Furthermore, the genetic and environmental components of skeletal malocclusion remain obscure.

**Figure 1:**
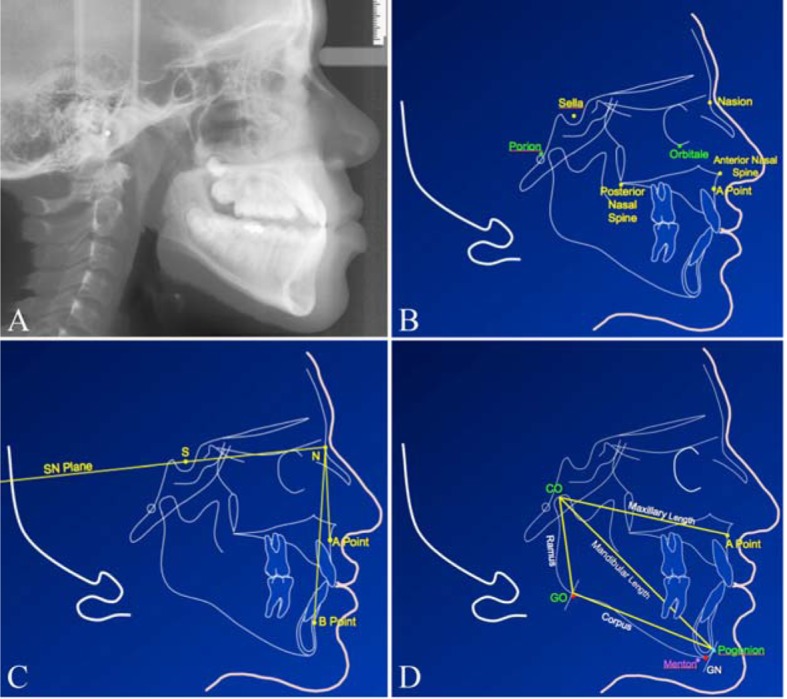
(A) Cephalometric radiograph of a 13-year-old male. (B) Cephalometric tracing of skeletal landmarks used for geometric constructions. (C) The anterior cranial base (S-N plane) and the angle between SNA and SNB planes that indicate the maxillary and mandibular positions, respectively. (D) The mandibular and maxillary landmarks and planes used to measure the length. Co-Pg is the linear mandibular length from condylion to pogonion. Co-Go is the ramus length from the condylion to the gonion. Go-Pg is the mandibular corpus length from the gonion to the pogonion.

Skeletal malocclusion according to Angle’s classification falls into class II and III depending on the position of the upper first molar to the lower first molar. In class II malocclusion, the mesiobuccal cusp of the upper first molar is mesially (anteriorly) positioned relative to the buccal groove of the lower first molar, while in class III, the mesiobuccal cusp of the upper first molar is distally (posteriorly) positioned relative to the buccal groove of lower first molar [6, 7]. A cephalometric radiograph for the face allows the construction of geometric cranial planes and the measurement of different lengths and angles of the jaws (Fig. 1A, B). Relating the position of the maxilla and mandible to the anterior cranial base is the most widely used method of assessing the anterior/posterior (AP) relationship of the maxilla and mandible. The line joining the midpoint of sella turcica (S) and the junction of the frontal and nasal bone (N) represent the position of the anterior cranial base (Fig. 1B). The deepest concavity of the maxillary (point A) and mandibular alveolar processes (point B) represent corresponding maxilla and mandible in the AP plane, and therefore the angle S-N-A represents the AP position of the maxilla in relation to the cranial base. Similarly the S-N-B angle represents the same for the mandible (Fig. 1C). Numerous studies have attempted to assess the “normal” values for SNA, SNB and other cephalometric variables. Surprisingly the norms for SNA and SNB in all these studies across the years are well established and very similar [4, 8]. In addition, they are universally used by orthodontists and oral and maxillofacial surgeons, and so there is no need for special radiographic investigations, as these data can be obtained from routine orthodontic and surgical records. Therefore, we suggest the use of SNA and SNB as a measure of retrognathisim and prognathisim (abnormal posterior or anterior position of the mandible or maxilla).

However, there are some limitations to this method that need to be addressed. The points A and B are not on the basal bone so they are affected by the movement of teeth. This should not be a problem in a cross-sectional and pre-treatment study. The other limitation is that SNA and SNB do not take the direction of growth into consideration. While this is not really a problem in the maxilla as it tends to grow downward and forward from the cranial base, mandibular growth can have one of two growth rotations: clockwise or anti-clockwise. The clockwise rotation brings the mandible downward and backward with growth that consequently makes retrognathia worse with respect to overall facial appearance. On the other hand, anti-clockwise rotation does the opposite (downwards and forwards) which improves retrognathia but makes prognathia worse. It is important to mention that the geometric values for normal occlusion vary between racial groups, genders and ages [9].

The difference between the lengths of the mandibular and maxillary in males and females is not significant at younger ages [7]. However, the differences become more prominent at the age of 12 years or older. To determine the severity of skeletal malocclusion, it is important to develop norm values for different racial groups and for males and females of each ethnicity. For measurement of mandibular micrognathia and macrognathia, we propose a linear measurement of the condylion (Co) to the pogonion (Pg) for the diagonal mandibular length, condylion (Co) to the gonion (Go) for the ramus length and gonion (Go) to pogonion (Pg) for the corpus length (Fig. 1D). To minimize the degree of error for clinical treatment of skeletal malocclusion, it is recommended to include the parents for craniofacial assessment and analysis because jaw phenotypes are heritable. Therefore, there is a big need for more research on skeletal malocclusion to identify genetic risk factors. Advanced research on this subject will help improve the precision of prognosis for therapeutic approaches.

Skeletal malocclusion affects dental and facial tissues as mentioned before. There are a handful of reports showing that skeletal malocclusion can affect the general health of patients through their role in causing airway obstructions, sleep apnea, gastric disturbance, immune deficiencies and delayed developmental growth [5, 6, 10]. Besides these physiological disorders, it has been reported that skeletal malocclusion leads to adverse influences on intellectual wellbeing, social skills, economical and psychological status [10, 11]. Psychological distress is more readily found to be associated with malocclusion especially in the younger and university educated people [10]. Severity of skeletal malocclusion is indirectly proportional to the quality of life in regards to social and emotional fronts as well as speech and mastication efficiency [10]. Bruxism, dental trauma and dental caries are significantly more prevalent in skeletal malocclusion cases compared to normal occlusion cases [12-14]. So far, little is known about the association between skeletal malocclusion and late onset diseases.

## Prevalence of Malocclusion

Prevalence of classes I and II malocclusion are very high in comparison to class III and other craniofacial anomalies. Lack of randomization of the data [15] and failure to include other races in the studies are the chief drawbacks of malocclusion classification. In 1959, Altemus reported a similar study in Black Americans for the first time. His results showed noticeable deviation from Angle’s work in 1907 (Table 1). Another study by Garner and Butt suggested a difference in prevalence among black Americans and Kenyan Population. The results for Black Americans were similar to the Altemis’s results. There were no class II div II cases found in Kenyan sample [16] (Table 1).

**Table 1:** Prevalence of Class I, II and III Malocclusion in Different Ethnic Groups

Author, year	Ethnicities	Sample size	Class I (a + b)** (%)	Class II (a + b)*** (%)	Class III (%)
Angle, 1907	Caucasians [17]	1,000	69	19 + 4	3.40
Altemus, 1959	Black American [18]	3,280	83	12	5
Cohen, 1970	Blacks/Whites [19]	410/349	71/53.6	11.4/33.6	6.3/4.7
Garner, 1985	Black American [16]	447	27 + 44	16	8.70
Garner, 1985	Kenyan [16]	471	16.8 + 51.7	7.90 + 0	16.80
Phaphe, 2012	Urban Indian [20]	1,000	18	30.1	1.60
Steigman, 1983	Israeli Arab [21]	803	85	8.5 + 1.7	1.3
Silva, 2001	Latino [22]	507	62.9 + 6.5	21.5	9.1
Lew, 1993	Chinese [23]	1,050	7.1 + 58.8	21.5	12.6
Garbin, 2010	Brazilian [24]	734	55.92	42.86	1.22
Hamdan, 2001	Jordanian [25]	320	62.5	21.5	16
Average			68.7	20.9	7.2

**Class I (a + b) = class I neutrocclusion + class I malocclusion. ***Class II (a + b) = class II Div I + class II Div II.

In 1983, Kapila reported that the frequency of normal occlusion was significantly more in African children compared to Asian children [16]. No gender difference was documented in all three classes of malocclusion. This fact was repeatedly backed by many studies [26-32]. Emrich, Brodie and Blayney indicated in a study conducted in 1965 that class II malocclusion was twice as more prevalent in white population compared to black population [15], while class III malocclusion was more frequent in blacks when compared with whites [29] (Table 1). Class III malocclusion is distributed heterogeneously in different races ranging from less than 5% in whites and up to 14% in Syrian natives [33]. Prevalence of class III malocclusion has been reported to be 12%, 10.5% and 9.4% in Asian, Caucasian Egyptians and Saudi Arabian population respectively [34]. Boeck et al [35] analyzed 171 cases with skeletal malocclusion and the classified them into the three different classes of malocclusion across different sexes and ethnicities. Table 2 shows the percentages of class I, class II and class III in 171 patients affected with skeletal malocclusion patients across sex and race [35].

**Table 2:** Percentages of Class I, Class II and Class III Cases With Skeletal Malocclusion Patients Across Sex and Race

	Class I (n = 11)	Class II (n = 79)	Class III (n = 81)
Sex			
Male	5.80%	36.20%	58%
Female	6.90%	52.90%	40.20%
Race			
Caucasian	6.30%	48.10%	45.60%
Black	10%	20%	70%

## The Etiology of Skeletal Malocclusion

About 65% of the US population has some degree of malocclusion; however, the cause of malocclusion for the vast majority of the cases is unknown. Children born with craniofacial disorders could be tested for genetic risk factors, but how this information can help in therapeutic approaches is not clear. Harris and Johnson in 1991 reported that the heritability of craniofacial features is very high in comparison to dental characteristics [36]. The pedigree of the Hapsburg Royal family with the prominent prognathic mandible strongly argues in favor of a genetic component in the inheritance of this craniofacial feature. It was noticed that 1/3 of the affected individuals of Hapsburg family with severe class III malocclusion had one parent with a similar phenotype. The pattern of inheritance in Hapsburg family indicate that the mandibular prognathia is not a Mendelian disorder but rather a multifactorial genetic disorder [3]. Patients with Angelman syndrome and fragile X-syndrome have prognathic mandible as a distinctive feature indicating a role of gene dosage and function in mandibular phenotype. Thus, for skeletal malocclusion therapy, if the parents of affected child are included for correlation and analysis, the percentage of successful prognosis is increased [37, 38].

## Risk Factors in Skeletal Malocclusion

Twin and consanguineous studies have confirmed that mandibular anomalies are commonly encountered genetic conditions [38-41]. These disorders can be studied prenatally but proper diagnosis is difficult as the isolated micrognathia is rarely (< 20%) present and other associated anomalies are challenging to interpret by prenatal examinations [5]. Although genetic involvement has more credit to itself, environmental components [42], like hormones, enlarged tonsils, trauma, instrumental deliveries, also contribute to the phenotype expression in malocclusion. This can be attributed to a notable hindrance in predicting the genetics of skeletal malocclusion [38]. It is almost impossible to control skeletal malocclusion by any kind of early orthodontic treatments due to genetic interference. Orthodontic treatments coupled with orthognathic surgery remain the only resort to overcome this problem [39]. Due to its multi-factorial etiology, some of the elements are possibly being controlled prenatally. In our parallel study, “Discovery of genetic risk factors in micrognathia, a manifestation of Class II skeletal malocclusion”, we aim to identify genetic risk factors involved in the nonsyndromic micrognathia and determine the prevalence of micrognathia and retrognathia in Houston. The results of this study will enhance our understanding of the genetic role, thereby providing us with better understanding for the pathophysiology of the craniofacial disorders and molecular targets for therapeutic approaches [40].

## Classification of Malocclusion for Research Purposes

Malocclusion is one of the most prevalent developmental anomalies of craniofacial structure, the subject of this review. Dr. Edward H. Angle is considered one of the pioneers in developing the field of orthodontics. Angle’s classification in 1899 of occlusion was an important step for formal diagnosis of malocclusion cases toward improving therapeutic approaches. The classification is based on the position of lower molar toward the upper molar and whether it is distally or mesially positioned. However, the distinction between dental vs. skeletal malocclusion for class II and III has not been clearly established. For the purposes of basic and translational research, it is important to determine the contribution and the severity of dental and skeletal malocclusion. Therefore, we propose a modified version of Angle’s classification to make this distinction clearer. The modified version is as follows: Class I: 1) neutrocclusion (ideal); 2) dental malocclusion (including bimaxillary protrusion, spacing, crowding, deep bite and open bite). Class II: 1) malocclusion without skeletal anomalies (first lower molar distally positioned); 2) mandibular retrognathia/micrognathia; 3) maxillary prognathia/macrognathia; 4) mandibular retrognathia and maxillary prognathia. Class III: 1) malocclusion without skeletal anomalies (first lower molar mesially positioned); 2) mandibular prognathism/macrognathia; 3) maxillary retrognathia/micrognathia; 4) mandibular prognathism and maxillary retrognathia.

According to a report published by the Center of Disease Control (CDC) in 1973 regarding the prevalence of malocclusion in US population, they classified the severity of malocclusion based on the treatment priority index (TPI). This index classifies the severity of malocclusion into five classes based on the need for a treatment (Table 3).

**Table 3:** The Frequencies of Various Occlusion/Malocclusion Categories in US Population Under Different Levels of TPI Scales

TPI scale	Category	Frequency (%)
0	Normal occlusion	24.4
1 - 3	Minor manifestations and treatment need is slight	39.0
4 - 6	Definite malocclusion but treatment elective	8.7
7 - 9	Severe handicap, treatment highly desirable	22.4
10	Very severe handicap with treatment mandatory	5.5

## Association Between Skeletal Malocclusion and Other Medical Conditions

Although skeletal malocclusion is responsible for several problems of oral cavity [11-14], few studies have discussed its association with other medical conditions and the impact on late-onset diseases.

### Dental anomalies

Skeletal malocclusion affects oral health quality and is highly associated with dental trauma and mastication difficulties as secondary effects of bruxism and teeth crowding [10, 14]. Bruxism is a parafunctional activity of oral cavity and is not related to eating or talking. It is characterized by excessive grinding of teeth or clenching of jaws. Bruxism may worsen malocclusion and leads to hypersensitive teeth, facial muscle pain and fatigue in almost all cases [13]. Patients with skeletal malocclusion are more likely to be presented with decayed, missed and filled teeth as compared to those with normal occlusion [10, 43]. A study focused on the prevalence of dental anomalies in a population of malocclusion patients found that at least one dental anomaly was present among 74.8% of the orthodontic patients [44]. In addition, a higher proportion of temporomandibular joint (TMJ) dysfunction was found in patients with class II skeletal malocclusion as reported by Simmons [45]. Skeletal malocclusion causes constant stress in the oral cavity leading to teeth clenching and abnormal contraction of muscles. This leads to other degenerative and systemic diseases [46]. On the other hand, many studies showed that malocclusion does not cause an increase in the prevelance of dental caries or TMJ dysfunction [47, 48]. Although premature contacts between teeth in malocclusion may contribute to bruxism, the relationship is not linear and far from being a cause and effect.

### Cleft palate

During embryonic development of oral cavity, the secondary palatal shelves and tongue grow parallel to each other in the oral cavity, with the tongue initially located between the two shelves. The secondary palatal shelves grow vertically toward the bottom of the mouth until the tongue drops down during the 7 - 8 weeks of gestation. This downward movement of tongue and rising of the shelves is facilitated by the forward and downward movement of the mandible, as it grows [49]. In cases of micrognathia and/or retrognathia, the fetal mandible fails to grow forward and downward leading to abnormal growth of tongue that prevents the elevation and fusion of the secondary palatal shelves. In a study assessing the outcome of prenatal sonographic diagnosis of isolated micrognathia (class II skeletal malocclusion), 93% of the infants also suffered from cleft palate and/or respiratory distress on neonatal examination [2]. Therefore, it is critical to determine whether patients with cleft palate have mandibular abnormalities or not. The proper and accurate diagnosis of the cause of palatal cleft will facilitate the identification of the genetic risk factors and assist in estimating the recurrence risk.

### Sleep apnea

Sleep-related breathing disorders were presented at higher frequency in abnormal jaw conditions including mandibular retrognathia, lateral cross-bite and increased overjet [50]. Airway obstruction in these patients is caused by posterior prolapse of the tongue and may lead to sleep apneas and chronic hypoxia. This condition if left untreated can complicate the survival of the affected child. If the child is able to survive, there is a high possibility of presence of neurological and cognitive delay, cardiopulmonary complications and behavioral issues [51]. Higher prevalence of narrower upper pharyngeal airways have been observed in cases with class II malocclusion involving vertical growth patterns of facial structure, when compared with controls bearing class I and class II malocclusion with normal growth pattern [52]. Class III malocclusion can be presented either with maxillary retrusion or with mandibular protrusion with almost similar frequencies [34, 53, 54]. Rapid maxillary expansion during childhood is a procedure to correct maxillary retrusion or hypoplastic maxilla. Maxillary expansion aids in rectification of nasal resistance among patients with respiratory problems [55]. This suggests the role of class III malocclusion (in case of maxillary retrusion) in causing anterior and posterior nasal obstructions. In addition, a recent study reported an enhanced pulmonary function and significantly better sleep quality following bimaxillary orthognathic surgery in skeletal malocclusion patients [56].

### Gastric disturbance

Although malocclusion is responsible for several problems of oral cavity [2, 27, 29], few studies have reported its association with the digestive system. A study conducted on 11 normal occlusion females and 11 malocclusion females noticed a significant relationship between malocclusion and gastric emptying function. This could be due to increased functional burden on stomach due to inferior activity of masticatory muscles in the malocclusion patients [57] causing inadequate mastication of food in the oral cavity.

### Immune system disorders, leukaemia and lymphoma

Immune system anomalies play an eminent role in congenital disorders. The association of facial birth defects in context with immunodeficiency [58] has been suggested in previous studies. A case report of two brothers, with unusual facial features including micrognathia along with other skeletal developmental problems, reported the presence of chemotactic defect and transient hypogammaglobulinemia [59]. Association of lymphoma and craniofacial deformities is not well documented. Lymphomas along with leukaemia and neuroblastoma are associated with Dubowitz syndrome. This syndrome is a congenital disorder affecting face development [60, 61]. Facial features are usually characterized by small round face with pointed retrognathic chin giving it a triangular shape, a broad, wide tipped nose, shallow supraorbital ridge with drooping eyelids, and posteriorly angulated low set ears [62]. In the same vein, Down syndrome is a common genetic disorder that has a significantly higher risk for leukaemia [63] and is characterized with craniofacial dysmorphology and maxillary deficiencies [64]. These syndromic disorders indicate the need for significant research focussing on craniofacial anomalies and its relevance with leukaemia and lymphoma.

### Other medical conditions

Eggleston stated malocclusion as a probable cause of hypertension in one of his publications [46]. Kilcoyne also stated that dental occlusion problems are a major cause of headache [65]. Similarly, class II division I malocclusion according to Angle’s classification was significantly associated with myopia [38, 66]. Degenerative diseases of heart, joints and teeth are all related to presence of constant distress. Skeletal malocclusion is the contributor of constant distress. Such a stress also leads to myofacial pain and hypertension as a consequence of lack of blood circulation caused by poor contraction of smooth muscles. The relation between malocclusion and presence of hypertension was demonstrated by an experiment using occlusal splint. In this experiment, the use of an occlusal splint helped alleviate the stressors contributed by presence of malocclusion and caused an immediate drop in blood pressure [46].

The data of 3,019 orthodontic patients with all classes of malocclusion from the BigMouth Dental Data Repository have been used to identify association between malocclusion and other selected medical conditions [67] (Table 4). The assessment of medical conditions was done based on the clinical forms filled out by the orthodontic patients upon the clinical visit.

**Table 4:** Association Between Malocclusion and Different Medical Conditions for 3,019 Orthodontic (Malocclusion) Patients From the BigMouth Dental Data Repository [67]

Medical conditions (variable)	Chi-square value	P value
Sleep apnea	0.7728	0.379
GI disorders	12.7649	0.000**
Lymphoma	2.6326	0.105
General dental problems	0.0169	0.896
Loose broken or missing filling	2.2985	0.129
Bruxism	0.2680	0.605
Cleft lip and palate	0.8272	0.363
Hypertension	26.8761	0.000**
Headache	311.1025	0.000**
Vision problems	2.9614	0.085
Functional pain/discomfort of oral cavity	318.2418	0.000**
Active tuberculosis	91.6427	0.000**

**Statistical significance.

## Limitations of Our Analysis and Future Studies

We used malocclusion data for our analysis and these data were not classified into dental and skeletal malocclusion and hence, analysis was done on all mixed cases of dental and skeletal malocclusion.

We used Pearson’s Chi-square analysis to compute individual probabilities for association with selected medical conditions. The P value < 0.01 is considered highly significant (Table 4).

Based on published data, isolated severe cases of micrognathia and prognathia of mandible are less frequent compared to the syndromic forms. This makes the study less efficient in concluding the contributing factors of underlying medical conditions [2].

## Conclusions

Further research, targeting the association of congenital developmental disorders of jaws with deleterious immune system diseases (like tuberculosis) and less severe immune reactions (like allergies), will augment advanced treatment options. Studies targeting skeletal malocclusion in particular are recommended to get more promising results for identifying causes (etiological risk factors) and epidemiological information rather than relying on signs and symptoms to enhance differential diagnosis and management. This work was supported by a start-up fund from UTHealth School of Dentistry to WF.
